# 1,3-Bis(1-phenyl­eth­yl)imidazolidine-2-thione

**DOI:** 10.1107/S160053681201224X

**Published:** 2012-03-24

**Authors:** M. Naveed Umar, M. Nawaz Tahir, Mohammad Shoaib, Akbar Ali, Imran Khan

**Affiliations:** a Department of Chemistry, University of Malakand, Pakistan; b University of Sargodha, Department of Physics, Sargodha, Pakistan; c Department of Pharmacy, University of Malakand, Pakistan; d Department of Biotechnology, University of Malakand, Pakistan

## Abstract

The complete molecule of the title compound, C_19_H_22_N_2_S, is generated by crystallographic twofold symmetry with the C=S group lying on the rotation axis. The imidazolidine ring adopts a flattened twist conformation. The dihedral angle between the asymmetric part of the imidazolidine-2-thione fragment and the benzene ring is 89.49 (17)°.

## Related literature

For a related structure, see: Umar *et al.* (2012 ▶).
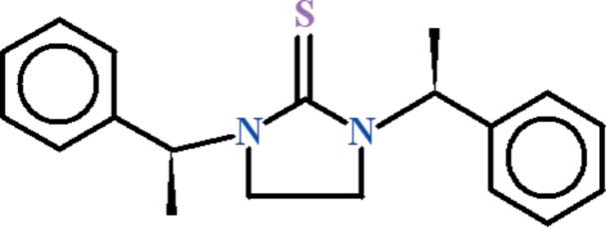


## Experimental

### 

#### Crystal data

C_19_H_22_N_2_S
*M* *_r_* = 310.45
Tetragonal, 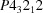

*a* = 5.8692 (5) Å
*c* = 50.637 (5) Å
*V* = 1744.3 (3) Å^3^
*Z* = 4
Mo *K*α radiation
μ = 0.18 mm^−1^
*T* = 296 K
0.28 × 0.24 × 0.20 mm

#### Data collection

Bruker Kappa APEXII CCD diffractometer
Absorption correction: multi-scan (*SADABS*; Bruker, 2005 ▶) *T*_min_ = 0.957, *T*_max_ = 0.966
18956 measured reflections
1717 independent reflections
1150 reflections with *I* > 2σ(*I*)
*R*_int_ = 0.062

#### Refinement

*R*[*F* ^2^ > 2σ(*F* ^2^)] = 0.070
*wR*(*F* ^2^) = 0.170
*S* = 1.11
1717 reflections
106 parameters
H atoms treated by a mixture of independent and constrained refinement
Δρ_max_ = 0.18 e Å^−3^
Δρ_min_ = −0.17 e Å^−3^
Absolute structure: Flack (1983 ▶), 569 Friedel pairs
Flack parameter: 0.1 (3)

### 

Data collection: *APEX2* (Bruker, 2007 ▶); cell refinement: *SAINT* (Bruker, 2007 ▶); data reduction: *SAINT*; program(s) used to solve structure: *SHELXS97* (Sheldrick, 2008 ▶); program(s) used to refine structure: *SHELXL97* (Sheldrick, 2008 ▶); molecular graphics: *ORTEP-3 for Windows* (Farrugia, 1997 ▶) and *PLATON* (Spek, 2009 ▶); software used to prepare material for publication: *WinGX* (Farrugia, 1999 ▶) and *PLATON*.

## Supplementary Material

Crystal structure: contains datablock(s) global, I. DOI: 10.1107/S160053681201224X/gk2471sup1.cif

Structure factors: contains datablock(s) I. DOI: 10.1107/S160053681201224X/gk2471Isup2.hkl

Supplementary material file. DOI: 10.1107/S160053681201224X/gk2471Isup3.cml

Additional supplementary materials:  crystallographic information; 3D view; checkCIF report

## Figures and Tables

**Fig. 1.**
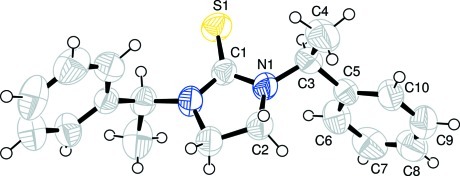
View of the title molecule with displacement ellipsoids drawn at the 50% probability level. H atoms are shown by small circles of arbitrary radii.

## supplementary crystallographic information

### Comment

The title compound, Fig. 1, has been synthesized as a part of our ongoing project related to imidazolidinethione.

Recently we have reported the crystal structure of 1,3-bis(1-cyclohexylethyl)imidazolidine (Umar *et al.*, 2012) that is related to the title compound.

The molecule has twofold rotation symmetry about the C=S bond of imidazolidinethione fragment and therefore the asymmetric unit consists of half of the molecule. The asymmetric part of imidazolidinethione fragment A (S1/C1/N1/C2) and the benzene ring B (C6/C7/C9/C10) form the dihedral angle of 89.49 (17)°.

### Experimental

(*S*)-1-Phenylethanamine (2.5 equiv.) and 1,2-dibromoethane (1 equiv.) were placed in a pressure vessel and heated at 393 K for 5 h, during which the reaction mixture solidified. The system was cooled to room temperature and NaOH (1 N, 20 ml) and ethyl acetate (20 ml) were added into the reaction mixture. After dissolving the reaction mixture, the crude product was extracted with ethyl acetate (3×25 ml). The combined organic layers were concentrated and subjected to column chromatography. The product obtained from column chromatography (1 equiv.) was added to toluene (0.4 *M*) in pressure vessel and thiocarbonyldiimidazol (1.1 equiv.) was added to it. This mixture was heated at about 373 K for 15 h. Again the extraction with ethyl acetate (3×25 ml) was carried out by using column chromatography to get the required product (yield: 80%).White prisms of of the title compound were obtained by recrystalization from methanol during 48 h (m.p. 416 K).

### Refinement

The H atoms were positioned geometrically (C–H = 0.93–0.98 Å) and refined as riding with *U*_iso_(H) = x*U*_eq_(C), where x = 1.5 for methyl and x = 1.2 for all other H-atoms.

### Crystal data

**Table:**

C_19_H_22_N_2_S	*D*_x_ = 1.182 Mg m^−^^3^
*M**_r_* = 310.45	Mo *K*α radiation, λ = 0.71073 Å
Tetragonal, *P*4_3_2_1_2	Cell parameters from 1150 reflections
Hall symbol: P 4nw 2abw	θ = 3.2–26.0°
*a* = 5.8692 (5) Å	µ = 0.18 mm^−^^1^
*c* = 50.637 (5) Å	*T* = 296 K
*V* = 1744.3 (3) Å^3^	Prism, white
*Z* = 4	0.28 × 0.24 × 0.20 mm
*F*(000) = 664	

### Data collection

**Table:**

Bruker Kappa APEXII CCD diffractometer	1717 independent reflections
Radiation source: fine-focus sealed tube	1150 reflections with *I* > 2σ(*I*)
Graphite monochromator	*R*_int_ = 0.062
Detector resolution: 7.80 pixels mm^-1^	θ_max_ = 26.0°, θ_min_ = 3.2°
ω scans	*h* = −3→7
Absorption correction: multi-scan (*SADABS*; Bruker, 2005)	*k* = −7→7
*T*_min_ = 0.957, *T*_max_ = 0.966	*l* = −62→62
18956 measured reflections	

### Refinement

**Table:**

Refinement on *F*^2^	Hydrogen site location: inferred from neighbouring sites
Least-squares matrix: full	H atoms treated by a mixture of independent and constrained refinement
*R*[*F*^2^ > 2σ(*F*^2^)] = 0.070	*w* = 1/[σ^2^(*F*_o_^2^) + (0.0459*P*)^2^ + 1.2139*P*] where *P* = (*F*_o_^2^ + 2*F*_c_^2^)/3
*wR*(*F*^2^) = 0.170	(Δ/σ)_max_ < 0.001
*S* = 1.11	Δρ_max_ = 0.18 e Å^−^^3^
1717 reflections	Δρ_min_ = −0.17 e Å^−^^3^
106 parameters	Extinction correction: *SHELXL97* (Sheldrick, 2008), Fc^*^=kFc[1+0.001xFc^2^λ^3^/sin(2θ)]^-1/4^
0 restraints	Extinction coefficient: 0.011 (3)
Primary atom site location: structure-invariant direct methods	Absolute structure: Flack (1983), 569 Friedel pairs
Secondary atom site location: difference Fourier map	Flack parameter: 0.1 (3)

### Special details

**Table:**

Geometry. All e.s.d.'s (except the e.s.d. in the dihedral angle between two l.s. planes) are estimated using the full covariance matrix. The cell e.s.d.'s are taken into account individually in the estimation of e.s.d.'s in distances, angles and torsion angles; correlations between e.s.d.'s in cell parameters are only used when they are defined by crystal symmetry. An approximate (isotropic) treatment of cell e.s.d.'s is used for estimating e.s.d.'s involving l.s. planes.
Refinement. Refinement of *F*^2^ against ALL reflections. The weighted *R*-factor *wR* and goodness of fit *S* are based on *F*^2^, conventional *R*-factors *R* are based on *F*, with *F* set to zero for negative *F*^2^. The threshold expression of *F*^2^ > σ(*F*^2^) is used only for calculating *R*-factors(gt) *etc*. and is not relevant to the choice of reflections for refinement. *R*-factors based on *F*^2^ are statistically about twice as large as those based on *F*, and *R*- factors based on ALL data will be even larger.

### Fractional atomic coordinates and isotropic or equivalent isotropic displacement parameters (Å^2^)

**Table:**

	*x*	*y*	*z*	*U*_iso_*/*U*_eq_	
S1	1.12952 (18)	1.12952 (18)	0.0000	0.0805 (6)	
N1	0.8146 (6)	0.8492 (6)	0.02136 (5)	0.0692 (10)	
C1	0.9276 (6)	0.9276 (6)	0.0000	0.0598 (14)	
C2	0.6343 (8)	0.6919 (7)	0.01411 (7)	0.0723 (12)	
H2A	0.4858	0.7636	0.0154	0.087*	
H2B	0.6367	0.5571	0.0252	0.087*	
C3	0.8267 (8)	0.9498 (8)	0.04762 (8)	0.0687 (12)	
H3	0.962 (7)	1.038 (7)	0.0469 (8)	0.082*	
C4	0.6154 (10)	1.0894 (8)	0.05338 (9)	0.1018 (18)	
H4A	0.5884	1.1932	0.0391	0.153*	
H4B	0.6372	1.1738	0.0694	0.153*	
H4C	0.4868	0.9897	0.0553	0.153*	
C5	0.8783 (7)	0.7647 (7)	0.06798 (7)	0.0579 (10)	
C6	1.0569 (8)	0.6131 (9)	0.06414 (9)	0.0843 (14)	
H6	1.1454	0.6219	0.0489	0.101*	
C7	1.1034 (9)	0.4468 (9)	0.08326 (11)	0.0963 (17)	
H7	1.2213	0.3437	0.0805	0.116*	
C8	0.9810 (11)	0.4343 (9)	0.10538 (10)	0.1002 (19)	
H8	1.0161	0.3253	0.1181	0.120*	
C9	0.8049 (10)	0.5808 (9)	0.10942 (9)	0.0949 (17)	
H9	0.7157	0.5700	0.1246	0.114*	
C10	0.7614 (8)	0.7429 (8)	0.09100 (7)	0.0764 (12)	
H10	0.6442	0.8456	0.0943	0.092*	

### Atomic displacement parameters (Å^2^)

**Table:**

	*U*^11^	*U*^22^	*U*^33^	*U*^12^	*U*^13^	*U*^23^
S1	0.0776 (8)	0.0776 (8)	0.0861 (11)	−0.0227 (10)	−0.0077 (7)	0.0077 (7)
N1	0.085 (3)	0.070 (2)	0.0521 (17)	−0.0229 (18)	−0.0062 (16)	0.0039 (17)
C1	0.062 (2)	0.062 (2)	0.056 (3)	−0.002 (3)	−0.007 (2)	0.007 (2)
C2	0.084 (3)	0.073 (3)	0.060 (2)	−0.024 (2)	−0.003 (2)	0.0031 (19)
C3	0.080 (3)	0.063 (3)	0.063 (2)	−0.005 (2)	−0.007 (2)	−0.006 (2)
C4	0.138 (5)	0.081 (4)	0.086 (3)	0.046 (4)	−0.015 (3)	−0.008 (3)
C5	0.057 (2)	0.063 (2)	0.053 (2)	0.001 (2)	−0.005 (2)	−0.0054 (18)
C6	0.070 (3)	0.108 (4)	0.074 (3)	0.012 (3)	0.003 (2)	−0.006 (3)
C7	0.089 (4)	0.090 (4)	0.110 (4)	0.037 (3)	−0.024 (3)	−0.012 (3)
C8	0.140 (5)	0.084 (4)	0.077 (3)	0.021 (4)	−0.034 (3)	−0.002 (3)
C9	0.123 (5)	0.097 (4)	0.065 (3)	0.006 (4)	0.003 (3)	0.006 (3)
C10	0.092 (3)	0.079 (3)	0.058 (2)	0.017 (2)	0.004 (2)	−0.001 (2)

### Geometric parameters (Å, º)

**Table:**

S1—C1	1.676 (5)	C4—H4C	0.9600
N1—C1	1.350 (4)	C5—C10	1.359 (5)
N1—C2	1.451 (5)	C5—C6	1.388 (6)
N1—C3	1.456 (5)	C6—C7	1.402 (7)
C1—N1^i^	1.350 (4)	C6—H6	0.9300
C2—C2^i^	1.507 (7)	C7—C8	1.333 (7)
C2—H2A	0.9700	C7—H7	0.9300
C2—H2B	0.9700	C8—C9	1.360 (7)
C3—C4	1.515 (6)	C8—H8	0.9300
C3—C5	1.528 (6)	C9—C10	1.357 (6)
C3—H3	0.95 (4)	C9—H9	0.9300
C4—H4A	0.9600	C10—H10	0.9300
C4—H4B	0.9600		
			
C1—N1—C2	111.9 (3)	C3—C4—H4C	109.5
C1—N1—C3	124.7 (3)	H4A—C4—H4C	109.5
C2—N1—C3	121.6 (3)	H4B—C4—H4C	109.5
N1^i^—C1—N1	107.9 (4)	C10—C5—C6	116.2 (4)
N1^i^—C1—S1	126.1 (2)	C10—C5—C3	123.1 (4)
N1—C1—S1	126.1 (2)	C6—C5—C3	120.7 (4)
N1—C2—C2^i^	102.7 (2)	C5—C6—C7	119.8 (4)
N1—C2—H2A	111.2	C5—C6—H6	120.1
C2^i^—C2—H2A	111.2	C7—C6—H6	120.1
N1—C2—H2B	111.2	C8—C7—C6	120.9 (5)
C2^i^—C2—H2B	111.2	C8—C7—H7	119.5
H2A—C2—H2B	109.1	C6—C7—H7	119.5
N1—C3—C4	110.8 (4)	C7—C8—C9	120.1 (5)
N1—C3—C5	109.7 (3)	C7—C8—H8	120.0
C4—C3—C5	114.6 (4)	C9—C8—H8	120.0
N1—C3—H3	103 (2)	C10—C9—C8	118.9 (5)
C4—C3—H3	113 (3)	C10—C9—H9	120.6
C5—C3—H3	104 (3)	C8—C9—H9	120.6
C3—C4—H4A	109.5	C9—C10—C5	124.1 (5)
C3—C4—H4B	109.5	C9—C10—H10	117.9
H4A—C4—H4B	109.5	C5—C10—H10	117.9
			
C2—N1—C1—N1^i^	−6.1 (2)	C4—C3—C5—C10	−7.4 (6)
C3—N1—C1—N1^i^	−171.0 (5)	N1—C3—C5—C6	49.8 (5)
C2—N1—C1—S1	173.9 (2)	C4—C3—C5—C6	175.2 (4)
C3—N1—C1—S1	9.0 (5)	C10—C5—C6—C7	1.4 (7)
C1—N1—C2—C2^i^	14.8 (5)	C3—C5—C6—C7	179.1 (4)
C3—N1—C2—C2^i^	−179.8 (4)	C5—C6—C7—C8	−1.2 (8)
C1—N1—C3—C4	102.8 (5)	C6—C7—C8—C9	1.5 (8)
C2—N1—C3—C4	−60.7 (5)	C7—C8—C9—C10	−2.0 (8)
C1—N1—C3—C5	−129.6 (4)	C8—C9—C10—C5	2.4 (8)
C2—N1—C3—C5	66.9 (5)	C6—C5—C10—C9	−2.1 (7)
N1—C3—C5—C10	−132.8 (4)	C3—C5—C10—C9	−179.7 (4)

Symmetry code: (i) *y*, *x*, −*z*.

### Hydrogen-bond geometry (Å, º)

**Table:**

*D*—H···*A*	*D*—H	H···*A*	*D*···*A*	*D*—H···*A*
C3—H3···S1	0.95 (4)	2.63 (4)	3.176 (4)	117 (3)
